# The diagnostic accuracy of pre-hospital assessment of acute respiratory failure

**DOI:** 10.29045/14784726.2020.12.5.3.15

**Published:** 2020-12-01

**Authors:** Gordon W Fuller, Steve Goodacre, Samuel Keating, Esther Herbert, Gavin Perkins, Matthew Ward, Andy Rosser, Imogen Gunson, Joshua Miller, Mike Bradburn, Tim Harris, Cindy Cooper

**Affiliations:** University of Sheffield: ORCID iD: https://orcid.org/0000-0001-8532-3500; University of Sheffield; University of Sheffield; University of Sheffield; University of Warwick; West Midlands Ambulance Service; West Midlands Ambulance Service; West Midlands Ambulance Service; West Midlands Ambulance Service; University of Sheffield; Barts and The London School of Medicine and Dentistry; University of Sheffield

**Keywords:** acute respiratory failure, diagnostic accuracy, emergency medical services, sensitivity, specificity

## Abstract

**Introduction:**

Acute respiratory failure (ARF) is a common medical emergency. Pre-hospital management includes controlled oxygen therapy, supplemented by specific management options directed at the underlying disease. The aim of the current study was to characterise the accuracy of paramedic diagnostic assessment in acute respiratory failure.

**Methods:**

A nested diagnostic accuracy and agreement study comparing pre-hospital clinical impression to the final hospital discharge diagnosis was conducted as part of the ACUTE (Ambulance CPAP: Use, Treatment effect and Economics) trial. Adults with suspected ARF were recruited from the UK West Midlands Ambulance Service. The pre-hospital clinical impression of the recruiting ambulance service clinician was prospectively recorded and compared to the final hospital diagnosis at 30 days. Agreement between pre-hospital and hospital diagnostic assessments was evaluated using raw agreement and Gwets AC1 coefficient.

**Results:**

77 participants were included. Chronic obstructive pulmonary disease (32.9%) and lower respiratory tract infection (32.9%) were the most frequently suspected primary pre-hospital diagnoses for ARF, with secondary contributory conditions recorded in 36 patients (46.8%). There was moderate agreement between the primary pre-hospital and hospital diagnoses, with raw agreement of 58.5% and a Gwets AC1 coefficient of 0.56 (95% CI 0.43 to 0.69). In five cases, a non-respiratory final diagnosis was present, including: myocardial infarction, ruptured abdominal aortic aneurysm, liver failure and sepsis.

**Conclusions:**

Pre-hospital assessment of ARF is challenging, with limited accuracy compared to the final hospital diagnosis. A syndromic approach, providing general supportive care, rather than a specifically disease-orientated treatment strategy, is likely to be most appropriate for the pre-hospital environment.

## Introduction

Acute respiratory failure (ARF) is a common medical emergency which occurs when heart or lung disease result in inadequate blood oxygen levels and/or increased blood carbon dioxide levels (Greene & Peters, 1994). It is caused by a number of common cardiac or respiratory diseases, including heart failure, pneumonia and exacerbations of chronic obstructive pulmonary disease (COPD) and asthma (Chapman, 1984). There are approximately 9000 ARF cases in England per year, with a high 14% risk of death within 30 days (Pandor et al., 2015). ARF has substantial health services costs, with patients often requiring prolonged hospital stays, ventilatory support and critical care admissions (Ray et al., 2006). ARF was responsible for over 3 million National Health Service (NHS) bed days in England in 2014 (Department of Health, 2014). Accurate diagnosis and optimised clinical management of ARF therefore have the potential to improve both health outcomes and cost effectiveness.

Current United Kingdom (UK) pre-hospital clinical practice guidelines recommend a standard management approach of oxygen therapy for the treatment of ARF, supplemented by specific management options directed at the underlying disease (AACE, 2019; NICE, 2010; Ponikowski et al., 2016). Pre-hospital administration of continuous positive airways pressure (CPAP) has been promoted as an additional potentially beneficial treatment strategy in some cases of ARF (Goodacre et al., 2014). An accurate pre-hospital diagnosis may help paramedics tailor therapy to the underlying cause of ARF and improve outcomes. Misdiagnosis could lead to inappropriate treatment, and even harm, for example instigating CPAP in patients with a pneumothorax (BTS, 2012; Davidson et al., 2016).

However, clinical assessment in the pre-hospital environment is often challenging. Details of previous medical history are often unavailable, dyspnoeic patients may not be able to provide a history, the uncontrolled environment can hamper examination, resuscitation of unstable patients may need to be prioritised and limited diagnostic tools are available. Furthermore, patients with ARF frequently suffer from multiple cardiorespiratory co-morbidities, or could have concurrent disease processes. There is limited data available investigating pre-hospital diagnosis of the dyspnoeic patient. The aim of the current study was to characterise the performance of paramedic clinical assessment in ARF. Specific objectives were to calculate diagnostic accuracy and agreement between pre-hospital and hospital diagnoses.

## Methods

A nested, pre-planned, diagnostic accuracy and agreement study, comparing pre-hospital clinical impression to the final hospital discharge diagnosis, was conducted as part of the ACUTE (Ambulance CPAP: Use, Treatment effect and economics) pilot trial. Study conduct and reporting was performed in accordance with STARD and GRRAS guidelines for diagnostic accuracy and reliability studies (Bossuyt et al., 2015; Kottner et al., 2011).

### Study population

The ACUTE trial was an individual patient randomised controlled external pilot trial to determine whether a definitive pragmatic randomised controlled trial (RCT) comparing pre-hospital CPAP to standard oxygen therapy for acute respiratory failure was feasible, acceptable and cost effective. The trial was pre-registered (ISRCTN12048261), and the protocol has been reported in detail previously (Fuller et al., 2018). Briefly, patients with suspected ARF were recruited from four ambulance hubs in the United Kingdom West Midlands Ambulance Service (WMAS) between August 2017 and July 2018. ARF was defined as respiratory distress with peripheral oxygen saturation below British Thoracic Society (BTS) target levels (88% for patients with COPD, or 94% for other conditions), despite supplemental oxygen (titrated low flow oxygen for COPD, or titrated high flow oxygen in other conditions; (O’Driscoll et al., 2017). Eligibility criteria are presented in Table 1. Patients were allocated to either pre-hospital CPAP (O_two system) with supplemental oxygen or standard oxygen therapy using identical equipment boxes (O_two CPAP unit, 2018), Feasibility outcomes were: incidence of recruited eligible patients (target 120); proportion recruited in error; adherence to the allocation schedule and treatment; and retention at 30 days. Effectiveness outcomes comprised: survival at 30 days; proportion undergoing endotracheal intubation; admission to critical care; and length of hospital stay.

**Table 1.** Eligibility criteria for the ACUTE trial.

Inclusion criteria	Exclusion criteria
Respiratory distress with peripheral oxygen saturations below: 88% for patients with COPD* 94% for other conditions* *Despite supplemental oxygen (titrated low flow oxygen for COPD, or titrated high flow oxygen in other conditions)	Hospital CPAP treatment available within 15 minutes of eligibility <18 years Terminal illness Pre-existing lack of capacity Documented not for resuscitation status Acutely incapacitated patients with known valid advanced directive declining non-invasive ventilation or participation in research Oxygen alert card Anticipated inability to apply CPAP (e.g. facial deformity) Respiratory failure due to chest trauma Contraindication to CPAP (suspected pneumothorax, respiratory arrest, epistaxis, vomiting, hypotension) Previous enrolment in the ACUTE trial Pregnancy Patients unable to communicate with ambulance service clinicians

### Data collection

A patient recruitment data collection form, contained within each equipment box, was completed by recruiting ambulance service clinicians every time a patient was enrolled in the trial. This recorded trial-specific information, including the pre-hospital clinical impression. At 30 days, research paramedics reviewed hospital records (including case notes, information systems and discharge letters), with patient consent, to collect details of clinical progress including the final medical diagnosis.

### Index test and reference standard

The index test under consideration was the trial paramedic’s clinical impression recorded at the scene of incident. Both the most likely clinical diagnosis and the presence of any contributing conditions were recorded prospectively by attending paramedics as a pre-specified six-category nominal variable, comprising: ‘heart failure’, ‘asthma’, ‘lower respiratory tract infection’, ‘chronic obstructive pulmonary disease’, ‘pulmonary embolism’ and ‘other’. These categories were chosen based on the most common causes of ARF, and conditions benefiting from specific treatment strategies. Conditions specified in the free text ‘other’ option were coded *post hoc* by two ACUTE co-investigators, with any disagreements resolved by discussion to achieve a consensus decision. The reference standard was the final hospital diagnosis accounting for the presenting respiratory distress provided by the hospital clinical team. Similarly to the index test, both the primary diagnosis and any contributory conditions were collected. These were determined retrospectively from the hospital case notes or discharge summary, and recorded using the same nominal categories by two ACUTE co-investigators. Hospital clinicians had access to routine pre-hospital patient records, but not the trial case report form containing the index test classification.

### Statistical analysis

The statistical analysis proceeded in three stages. Firstly, sample characteristics were described using summary statistics, cross tabulation and a mosaic plot. Secondly, agreement between pre-hospital and hospital diagnostic assessments was evaluated (Gwet, 2001; Kottner & Streiner, 2011). Raw agreement was initially calculated as the proportion of cases with an identical pre-hospital and hospital diagnosis (Gwet, 2008; Kottner et al., 2011). To account for the possibility that some agreement might be expected due to chance, the Gwets AC1 coefficient was also determined (Gwet, 2008). This statistic was chosen in preference to Cohen’s Kappa statistic as it does not depend upon an assumption of independence between different ratings, is robust to marginal probabilities and is less affected by rating prevalence (Wongpakaran et al., 2013). Landis and Koch’s benchmark values were chosen as the most established thresholds to interpret the magnitude of agreement coefficients with: 0–0.20 indicating slight, 0.21–0.40 fair, 0.41–0.60 moderate, 0.61–0.80 substantial and 0.81–1 almost perfect agreement (Landis & Koch, 1977). Agreement was calculated for the primary diagnoses alone; and for combined primary and secondary diagnoses, ignoring the precedence placed on each condition and counting any match. Thirdly, the pre-hospital primary clinical impressions (index tests) were compared to the final hospital diagnosis (reference standard), with sensitivity and specificity calculated for the most common diagnostic categories. All results were calculated with their 95% confidence intervals. Complete case analyses were conducted, with missing or non-interpretable data highlighted where relevant. As a pre-specified trial sub-study, a power calculation was not performed, and confidence interval width indicates the precision of results. Statistical analyses were carried out in R (R Foundation for Statistical Computing, Vienna, Austria) and AgreeStat 2011.3 (advanced Analytics, Gaithersburg, MD, USA).

### Ethics and funding

If possible, verbal consent was obtained for enrolment in the ACUTE trial at the scene of incident, with subsequent written informed consent confirmed for further participation. Patients lacking capacity were enrolled according to a hierarchical consent process complying with the English Mental Capacity Act 2005 (Johnston & Liddle, 2007). Ethical approval was confirmed with the NHS Leeds East Research Ethics Committee (31 October 2016, reference 16/YH/0406). The University of Sheffield provided sponsorship and monitoring oversight of the project. Funding was provided by the National Institute for Health Research’s HTA Programme (HTA Project: 15/08/40).

## Results

### Study sample

Over the trial recruitment period, 77 participants were enrolled from 364 potentially eligible patients by 41 individual ambulance service clinicians. Slightly more participants were allocated to the CPAP intervention arm (42 cases) than to the standard oxygen control arm (35 cases). Included patients were predominantly older (median 71 years), male (62%) and with marked respiratory distress (median oxygen saturations 78.5%, respiratory rate 34 breaths/minute and breathlessness score of 9/10). Patient characteristics of enrolled patients are summarised in Table 2. A valid pre-hospital primary diagnosis was available for 76/77 patients. In one case, the primary clinical impression was recorded as ‘other’, but lacked interpretable information to assign an underlying aetiology for ARF. A final hospital primary diagnosis was available for 65 patients who were included in the complete case agreement and diagnostic accuracy analyses (Table 2). Consent was declined for data collection in nine cases, clinical records were unavailable in two cases and in one case there was no clear underlying diagnosis apparent in the notes.

**Table 2.** Baseline characteristics of ACUTE trial participants.

Baseline variable	Descriptive statistic	All	Included	Excluded
		N = 77	N = 65	N = 12
Age	n	77	65	12
	Median (IQR)	71.00 (62.00, 77.00)	70.00 (62.00, 77.00)	73.50 (66.75, 86.00)
Sex	Male	48 (62.3%)	42 (64.6%)	6 (50.0%)
	Female	29 (37.7%)	23 (35.4%)	6 (50.0%)
Ancillary disease-specific pre-hospital treatments delivered	Yes	61 (79.2%)	53 (81.5%)	8 (66.7%)
	No	16 (20.8%)	12 (18.5%)	4 (33.3%)
Clinician’s assessment of patient's breathlessness at enrolment (VAS 0-10)	n	76	64	12
	Median (IQR)	9.00 (8.00, 10.00)	9.00 (8.00, 10.00)	8.00 (7.00, 10.00)
First systolic blood pressure (mmHg)	n	70	59	11
	Median (IQR)	134.50 (112.25, 152.00)	136.00 (109.50, 152.00)	127.00 (117.50, 135.50)
First Glasgow Coma Score	n	77	65	12
	Median (IQR)	15.00 (14.00, 15.00)	15.00 (14.00, 15.00)	15.00 (15.00, 15.00)
First oxygen saturations (%)	n	76	64	12
	Median (IQR)	78.50 (74.75, 86.00)	78.50 (74.00, 87.00)	79.50 (75.00, 82.25)
First pulse rate (bpm)	n	75	63	12
	Median (IQR)	115.00 (100.00, 124.00)	115.00 (95.50, 124.50)	114.00 (102.25, 124.00)
First respiratory rate (breaths/min)	n	77	65	12
	Median (IQR)	34.00 (28.00, 40.00)	34.00 (28.00, 40.00)	36.00 (27.00, 37.00)

IQR: Interquartile range; bpm: beats per minute; COPD: Chronic Obstructive Pulmonary Disease; LRTI: Lower Respiratory Tract Infection.

### Pre-hospital diagnosis

COPD (n = 25/76, 32.9%) and LRTI (n = 25/76, 32.9%) were the most commonly suspected primary pre-hospital diagnoses. In six cases (n = 76, 7.9%), a non-respiratory primary diagnosis was recorded, comprising: ruptured abdominal aortic aneurysm (n = 1), liver failure (n = 1), sepsis (not specified further, n = 2) and urinary tract infection (n = 2). A secondary diagnosis was recorded for 36 patients (n = 77, 46.8%), with a single contributory condition suspected in 29 patients (n = 77, 37.7%) and two supplementary diagnoses made for seven patients (n = 77, 9.1%). LRTI (n = 9/77, 11.7%) and heart failure (n = 10/77, 13.0%) were the most common concomitantly diagnosed conditions.

### Hospital diagnosis

The most common final diagnoses were COPD (21/65, 32.3%) and LRTI (n = 28/65, 43.1%). In four cases, a non-respiratory final diagnosis was given, including: myocardial infarction, ruptured abdominal, liver failure and sepsis (not specified further). Secondary conditions accounting for ARF were diagnosed in 27 patients (n = 65, 41.5%), with one additional condition recorded for 23 cases, two contributory diseases given for three cases and three further supporting diagnoses for one case. The commonest secondary diagnoses were COPD (n = 7/65, 10.8%) and heart failure (n = 8/65, 12.3%). Notably, two patients were diagnosed with a pneumothorax in hospital (one primary diagnosis, one secondary diagnosis, both requiring intercostal drains). Pre-hospital and final hospital diagnoses are summarised in Table 3.

**Table 3.** Pre-hospital and hospital ARF diagnosis.

		Total	Notes
Primary pre-hospital ARF diagnosis (n = 76)	COPD	25 (32.9%)	
	LRTI	25 (32.9%)	
	Heart failure	14 (18.4%)	
	Asthma	4 (5.3%)	
	Pulmonary fibrosis	2 (2.6%)	
	Other	6 (7.8%)	Sepsis (2); AAA (1); Liver failure (1); UTI (2)
Secondary contributory ARF pre-hospital diagnoses (n = 77)**	Present	36 (46.8%)	
	COPD	7 (9.1%)	
	LRTI	9 (11.7%)	
	Heart failure	10 (13.0%)	
	Asthma	5 (6.5%)	
	Pulmonary fibrosis	1 (1.3)	
	Other	6 (7.8%)	PE (1); Sepsis (1); Myocardial infarction (1); Pericarditis (1); Guilain-barre syndrome (1); Overdose (1)
Primary final hospital ARF diagnosis (n = 65)	COPD	21 (32.3%)	
	LRTI	28 (43.1%)	
	Heart failure	6 (9.2%)	
	Asthma	2 (3.1%)	
	Pulmonary fibrosis	1 (1.5%)	
	Other	7 (10.8%)	Sepsis (1); PE (1); AAA (1); Liver failure (1); Lung cancer (1); Myocardial Infarction (1); Pneumothorax (1)
Secondary contributory ARF final hospital diagnoses (n = 65)**	Present	27 (41.5%)	
	COPD	7 (10.8%)	
	LRTI	4 (6.2%)	
	Heart failure	8 (12.3%)	
	Asthma	3 (4.6%)	
	Pulmonary fibrosis	1 (1.5%)	
	Other	9 (13.8%)	Sepsis (2); Lung Cancer (2); Bronchiectasis (1); Pneumothorax (1); Morbid obesity (2); Anaemia (1)

** More than one secondary contributory diagnosis possible. COPD: Chronic Obstructive Pulmonary Disease; PE: Pulmonary Embolism; LRTI: Lower Respiratory Tract Infection; AAA: Abdominal Aortic Aneurysm; UTI: Urinary Tract Infection.

### Agreement

**Figure 1:**
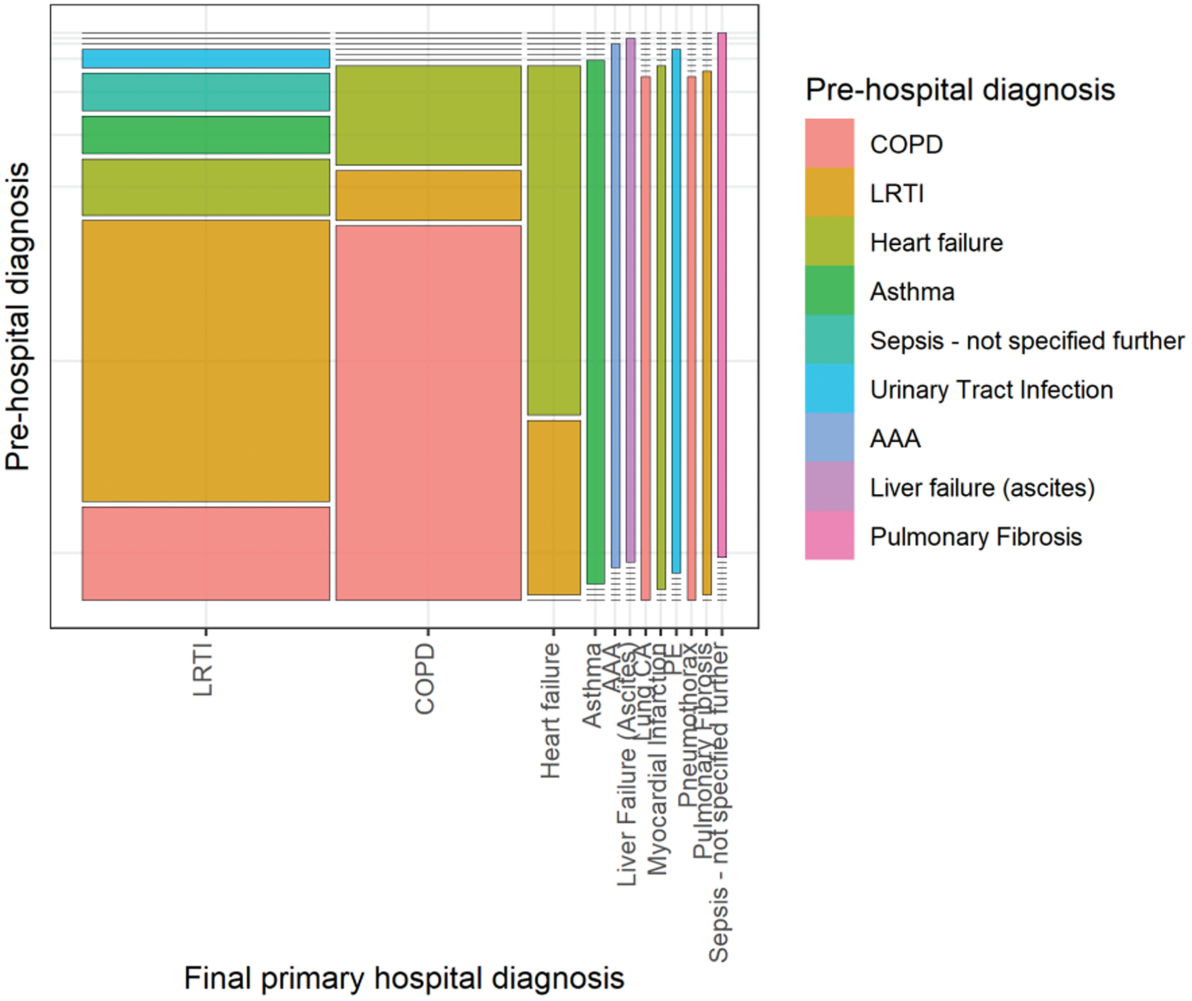
Figure 1. Mosaic plot demonstrating agreement between pre-hospital and hospital diagnoses.

There was limited reproducibility between the primary pre-hospital and hospital diagnoses, with raw agreement of 58.5% (n = 38/65). However, if both primary and secondary diagnoses were considered together, counting any match and ignoring the precedence placed on each condition, there was higher raw agreement of 76.9% on at least one causative disease for ARF (n = 50/65). Chance-corrected agreement between pre-hospital and hospital primary diagnosis was moderate, as demonstrated by a Gwets AC1 coefficient of 0.56 (95% CI 0.43 to 0.69). When both primary and secondary diagnoses were assessed together, there was substantial chance-corrected agreement on at least one condition, with a Gwets AC1 coefficient of 0.75 (95% CI 0.64 to 0.87). Agreement between pre-hospital and hospital diagnoses is presented in a mosaic plot in Figure 1 and is tabulated in the web appendix.

### Diagnostic accuracy

The performance of ambulance service clinicians’ assessment was then investigated by calculating diagnostic accuracy metrics for the most prevalent conditions (COPD, LRTI and heart failure). Other conditions were not evaluated due to low sample size, with consequent imprecision and intractability. While each condition was identified more correctly than not, all three were commonly missed as the primary diagnosis: the sensitivities for COPD, LRTI and heart failure were 71% (95% CI 48% to 89%), 54% (34% to 73%) and 67% (22% to 96%) respectively. The specificity was higher (COPD 84.1% (69.9% to 93.4%), LRTI 86.5% (71.2% to 95.5%) and heart failure 86.4% (75.0% to 94.0%)). When both primary and secondary diagnoses were assessed together, diagnostic accuracy was improved. Considering the index test and reference standard to be positive if the condition was recorded in either the primary or secondary diagnosis gave sensitivities of COPD 95.2%; LRTI 69.2%; Heart failure 85.7%, meaning all three conditions were typically identified, even if not as the primary diagnosis. Specificities in this contingency were: COPD 84.1%; LRTI 92.3%; Heart failure 96.6%.

## Discussion

COPD (n = 25/76, 32.9%) and LRTI (n = 25/76, 32.9%) were the most frequently suspected primary pre-hospital diagnoses for ARF, with secondary contributory conditions recorded in 36 patients (n = 77, 46.8%). There was moderate agreement between the primary pre-hospital and hospital diagnoses, with raw agreement of 58.5% (n = 38/65) and a Gwets AC1 coefficient of 0.56 (95% CI 0.43 to 0.69). In seven cases, a final diagnosis was present where CPAP would not be expected to be effective, or could be harmful, including: myocardial infarction, ruptured abdominal aortic aneurysm, liver failure, sepsis and pneumothorax (n = 7/65, 10.8%).

Respiratory distress with low oxygen saturations is common to many conditions, with symptoms and clinical signs shared between differential diagnoses, often making assessment challenging (Chapman, 1984; Delerme & Ray, 2008). It is therefore unsurprising that accuracy of the pre-hospital clinical impression was limited, non-specific working diagnoses such as ‘sepsis’ were used, some non-cardiorespiratory conditions were diagnosed and concurrent disease processes were suspected in the majority of cases. COPD and an LRTI were the most commonly diagnosed conditions, and distinction between these two entities is known to be difficult, even in hospital with the benefit of time, access to testing and specialist review (Finney et al., 2019).

Given that the most important treatment for ARF is provision of oxygen, and other treatment modalities currently available to UK paramedics (e.g. nebulisers) have few side effects, it could be argued that an exact pre-hospital diagnosis is unnecessary prior to definitive hospital care (Chapman, 1984). However, if CPAP or non-invasive ventilation is available, then it is important to recognise conditions representing relative or absolute contraindications (Hess, 2013). Although low numbers of patients were studied, it is reassuring that all cases with a final diagnosis of asthma were recognised by paramedics, but potentially concerning that there were two patients with undetected pneumothorax.

This is the first study to investigate the diagnostic assessment of patients with ARF presenting to EMS. Previous literature has either focused on less unwell dyspnoeic patients or examined specific diseases including COPD, asthma or heart failure (Christie et al., 2016; Williams et al., 2013, 2015). Although limited by retrospective chart review designs, this body of research has demonstrated similar findings to the current study. Christie et al. reported only moderate agreement between paramedic and hospital diagnosis in a New Zealand cohort, with many cases having no clearly documented working diagnosis (Christie et al., 2016). The sensitivity for pre-hospital heart failure, asthma and COPD diagnoses was only 29%, 66% and 39% respectively, in Australian EMS studies by Williams and colleagues (Williams et al., 2013, 2015). More widely, a recent systematic review reported a pooled sensitivity of 0.74 (0.62 to 0.82) and a pooled specificity of 0.94 (0.87 to 0.97) for paramedic diagnosis of myocardial infarction, sepsis, stroke or all diagnoses (Wilson et al., 2018).

Participating WMAS ambulance hubs serve a mixed rural, semi-rural and urban population, and the sample of recruited ARF patients should ensure good external validity to similar EMS settings. However, clinical trial populations may not be fully representative of undifferentiated pre-hospital ARF patients after application of eligibility criteria and consent procedures. The ACUTE trial specifically excluded patients with pre-existing lack of capacity or inability to communicate with trial paramedics – groups for which pre-hospital diagnosis was likely to be even more challenging. Generalisability to areas in which disease prevalence differs to the UK, or which have alternative EMS models (e.g. physician rather than paramedic assessment), is less certain.

The prospective, preplanned data collection, using a defined nominal categorisation for ARF, is a strength of this study. However, there are a number of limitations that could adversely affect the internal validity of the results. Firstly, there is the potential for reference standard misclassification, as the final diagnosis was recorded from the hospital record or discharge letter, rather than determination through formal expert case review. Secondly, although comparing favourably to other published reproducibility studies, the sample size is relatively low, resulting in imprecise results consistent with either poor or moderate agreement. The sample size constraint also meant that we did not attempt to model any clustering for differential effects of paramedics. These findings should therefore be considered as exploratory, requiring confirmation in larger studies. Thirdly, mainly due lack of consent, some reference standard data were missing. Although this represented a relatively small number (<10%) of patients, with similar characteristics to included cases, selection bias is possible if excluded patients differed systematically from the study population. Finally, we pre-specified the relatively liberal Landis and Koch scale for benchmarking agreement coefficients. Although well established and widely used, this may overstate agreement compared to other benchmarks, e.g. Fleiss’ or McHugh’s proposed scales (Fleiss et al., 2003; McHugh, 2012).

In conclusion, pre-hospital assessment of ARF is challenging, with limited diagnostic accuracy compared to the final hospital diagnosis. A syndromic approach, providing general supportive care, rather than a specifically disease-orientated treatment strategy, is likely to be most appropriate for ARF in the pre-hospital environment.

## Author contributions

GF and SG conceived and designed the study. AR, MW, IG and JM collected data. All authors were involved in the analysis and the interpretation of data. GF drafted the report. All authors revised the work critically for important intellectual content and were involved in the final approval of the version to be published. All authors agree to be accountable for all aspects of the work. SG acts as the guarantor for this article.

## Conflict of interest

Professor Steve Goodacre is Deputy Director of the NIHR HTA Programme, Chair of the NIHR HTA Commissioning Board and member of the NIHR HTA Funding Strategy Group. Professor Gavin Perkins is an NIHR Senior Investigator and member of the Programme Grants for Applied Research Board. Professor Cindy Cooper is a member of the NIHR Clinical Trials Unit Standing Advisory Committee and of the UK Clinical Research Collaboration Registered Clinical Trials Unit Network Executive Group.

## Ethics

Ethical approval was confirmed with NHS Leeds East Research Ethics Committee. The University of Sheffield provided sponsorship and monitoring oversight of the project.

## Funding

Funding was provided by the National Institute for Health Research’s HTA Programme (HTA Project: 15/08/40).

## References

[bibr_1] AACE. (2019). *UK ambulance services clinical practice guidelines: 2019.* Class Professional Publishing. Association of Ambulance Chief Executives.

[bibr_2] Bossuyt P. M. Reitsma J. B. Bruns D. E. Gatsonis C. A. Glasziou P. P. Irwig L. Lijmer J. G. Moher D. Rennie D. de Vet H. C. Kressel H. Y. Rifai N. Golub R. M. Altman D. G. Hooft L Korevaar D. A. & Cohen J. F. (2015). STARD 2015: An updated list of essential items for reporting diagnostic accuracy studies. *BMJ*, 351, h5527. 26511519 10.1136/bmj.h5527 PMC4623764

[bibr_3] BTS. (2012). *National respiratory audit programme annual report 2011/2012*. British Thoracic Society

[bibr_4] Chapman G. W. (1984). Pathophysiology and treatment of acute respiratory failure. *Journal of the National Medical Association*, 76(2), 201–206. 6708127 PMC2561752

[bibr_5] Christie A. Costa-Scorse B. Nicholls M. Jones P. & Howie G. (2016). Accuracy of working diagnosis by paramedics for patients presenting with dyspnoea. *Emergency Medicine Australasia*, 28(5), 525–530. 27397643 10.1111/1742-6723.12618

[bibr_6] Davidson C. Banham S. Elliott M. Kennedy D. Gelder C. Glossop A. Church C. Creagh-Brown B. Dodd J. Felton T. Foëx B. Mansfield L. McDonnell L. Parker R. Patterson C. Sovani M. & Thomas L. (2016). British Thoracic Society/Intensive Care Society guideline for the ventilatory management of acute hypercapnic respiratory failure in adults. *BMJ Open Respiratory Research*, 3(1), e000133–e000133. 27026806 10.1136/bmjresp-2016-000133 PMC4800170

[bibr_7] Delerme S. & Ray P. (2008). Acute respiratory failure in the elderly: Diagnosis and prognosis. *Age and Ageing*, 37(3), 251–257. 18388161 10.1093/ageing/afn060

[bibr_8] Department of Health. (2014). *Hospital episode statistics*. Retrieved August 20, 2020, from http://www.hesonline.nhs.uk.

[bibr_9] Finney L. J. Padmanaban V. Todd S. Ahmed N. Elkin Sarah L. & Mallia P. (2019). Validity of the diagnosis of pneumonia in hospitalised patients with COPD. *ERJ Open Research*, 5(2), 00031–02019. 31249841 10.1183/23120541.00031-2019 PMC6589445

[bibr_10] Fleiss J. L. Levin B. A. & Paik M. C. (Eds). (2003). *Statistical methods for rates and proportions* (3rd ed.). Wiley-Interscience.

[bibr_11] Fuller G. W. Goodacre S. Keating S. Perkins G. Ward M. Rosser A. Gunson I. Miller J. Bradburn M. Thokala P. Harris T. Carson A. Marsh M. & Cooper C. (2018). The ACUTE (Ambulance CPAP: Use, Treatment effect and economics) feasibility study: A pilot randomised controlled trial of pre-hospital CPAP for acute respiratory failure. *Pilot and Feasibility Studies*, 4, 86–86. 29946477 10.1186/s40814-018-0281-9 PMC6004668

[bibr_12] Goodacre S. Stevens J. W. Pandor A. Poku E. Ren S. Cantrell A. Bounes V. Mas A. Payen D. Petrie D. Roessler M. S. Weitz G. Ducros L. & Plaisance P. (2014). Pre-hospital noninvasive ventilation for acute respiratory failure: Systematic review, network meta-analysis, and individual patient data meta-analysis. *Academic Emergency Medicine*, 21(9), 960–970. 25269576 10.1111/acem.12466

[bibr_13] Greene K. E. & Peters J. I. (1994). Pathophysiology of acute respiratory failure. *Clinics in Chest Medicine*, 15(1), 1–12. 8200186

[bibr_14] Gwet K. (2001). *Handbook of inter-rater reliability: How to estimate the level of agreement between two or multiple raters.* STATAXIS Publishing Company.

[bibr_15] Gwet K. L. (2008). Computing inter-rater reliability and its variance in the presence of high agreement. *British Journal of Mathematical and Statistical Psychology*, 61(Pt 1), 29–48. 18482474 10.1348/000711006X126600

[bibr_16] Hess D. R. (2013). Noninvasive ventilation for acute respiratory failure. *Respiratory Care*, 58(6), 950–972. 23709194 10.4187/respcare.02319

[bibr_17] Johnston C. & Liddle J. (2007). The Mental Capacity Act 2005: A new framework for healthcare decision making. *Journal of Medical Ethics*, 33(2), 94–97. 17264196 10.1136/jme.2006.016972 PMC2598235

[bibr_18] Kottner J. Audige L. Brorson S. Donner A. Gajewski B. J. Hrobjartsson A. & Streiner D. L. (2011). Guidelines for Reporting Reliability and Agreement Studies (GRRAS) were proposed. *Journal of Clinical Epidemiology*, 64(1), 96–106. 21130355 10.1016/j.jclinepi.2010.03.002

[bibr_19] Kottner J. & Streiner D. L. (2011). The difference between reliability and agreement. *Journal of Clinical Epidemiology*, 64(6), 701–702. 21411278 10.1016/j.jclinepi.2010.12.001

[bibr_20] Landis J. R. & Koch G. G. (1977). The measurement of observer agreement for categorical data. *Biometrics*, 33(1), 159–174. 843571

[bibr_21] McHugh M. L. (2012). Interrater reliability: The kappa statistic. *Biochemia Medica: Casopis Hrvatskoga Drustva Medicinskih Biokemicara*, 22(3), 276–282. PMC3900052 23092060

[bibr_22] NICE. (2010). *Chronic obstructive pulmonary disease in over 16s: Diagnosis and management Clinical guideline [CG101]*. National Institute for Health and Care Excellence. 31211541

[bibr_23] O‘Driscoll B. R. Howard L. S. Earis J. & Mak V. (2017). BTS guideline for oxygen use in adults in healthcare and emergency settings. *Thorax*, 72(Suppl 1), ii1–ii90. 10.1136/thoraxjnl-2016-209729 28507176

[bibr_24] O_two CPAP unit. (2018). Retrieved Janury 10, 2020, from http://otwo.com/emergency-cpap/o_two-single-use-cpap/.

[bibr_25] Pandor A. Thokala P. Goodacre S. Poku E. Stevens J. W. Ren S. Cantrell A. Perkins G. D. Ward M. & Penn-Ashman J. (2015). Pre-hospital non-invasive ventilation for acute respiratory failure: A systematic review and cost-effectiveness evaluation. *Health Technology Assessment*, 19(42), v–vi, 1–102. 10.3310/hta19420 PMC4781299 26102313

[bibr_26] Ponikowski P. Voors A. A. Anker S. D. Bueno H. Cleland J. G. Coats A. J. Falk V. González-Juanatey J. R. Harjola V. P. Jankowska E. A. Jessup M. Linde C. Nihoyannopoulos P. Parissis J. T. Pieske B. Riley J. P. Rosano G. M. C. Ruilope L. M. Ruschitzka F. . . . & van der Meer P. (2016). 2016 ESC Guidelines for the diagnosis and treatment of acute and chronic heart failure: The Task Force for the diagnosis and treatment of acute and chronic heart failure of the European Society of Cardiology (ESC). Developed with the special contribution of the Heart Failure Association (HFA) of the ESC. *European Journal of Heart Failure*, 18(8), 891–975. 27207191 10.1002/ejhf.592

[bibr_27] Ray P. Birolleau S. Lefort Y. Becquemin M. H. Beigelman C. Isnard R. Teixeira A. Arthaud M. Riou B. & Boddaert J. (2006). Acute respiratory failure in the elderly: Etiology, emergency diagnosis and prognosis. *Critical Care (London, England)*, 10(3), R82. 16723034 10.1186/cc4926 PMC1550946

[bibr_28] Williams T. A. Finn J. Celenza A. Teng T. H. & Jacobs I. G. (2013). Paramedic identification of acute pulmonary edema in a metropolitan ambulance service. *Pre-hospital Emergency Care*, 17(3), 339–347. 10.3109/10903127.2013.773114 23484502

[bibr_29] Williams T. A. Finn J. Fatovich D. Perkins G. D. Summers Q. & Jacobs I. (2015). Paramedic differentiation of asthma and COPD in the pre-hospital setting is difficult. *Pre-hospital Emergency Care*, 19(4), 535–543. 10.3109/10903127.2014.995841 25664482

[bibr_30] Wilson C. Harley C. & Steels S. (2018) Systematic review and meta-analysis of pre-hospital diagnostic accuracy studies. *Emergency Medicine Journal,* 35(12), 757–764. 30217952 10.1136/emermed-2018-207588

[bibr_31] Wongpakaran N. Wongpakaran T. Wedding D. & Gwet K.L. (2013). A comparison of Cohen’s Kappa and Gwet’s AC1 when calculating inter-rater reliability coefficients: A study conducted with personality disorder samples. *BMC Med Res Methodol*, 13, 61. 23627889 10.1186/1471-2288-13-61 PMC3643869

